# Intra-Abdominal Candidiasis: The Importance of Early Source Control and Antifungal Treatment

**DOI:** 10.1371/journal.pone.0153247

**Published:** 2016-04-28

**Authors:** Pascalis Vergidis, Cornelius J. Clancy, Ryan K. Shields, Seo Young Park, Brett N. Wildfeuer, Richard L. Simmons, M. Hong Nguyen

**Affiliations:** 1 Department of Medicine, Division of Infectious Diseases, University of Pittsburgh School of Medicine, Pittsburgh, PA, United States of America; 2 VA Pittsburgh Healthcare System, Pittsburgh, PA, United States of America; 3 Department of Medicine, University of Pittsburgh School of Medicine, Pittsburgh, PA, United States of America; 4 Department of Surgery, University of Pittsburgh School of Medicine, Pittsburgh, PA, United States of America; University of Wisconsin Medical School, UNITED STATES

## Abstract

Intra-abdominal candidiasis (IAC) is poorly understood compared to candidemia. We described the clinical characteristics, microbiology, treatment and outcomes of IAC, and identified risk factors for mortality. We performed a retrospective study of adults diagnosed with IAC at our center in 2012–2013. Risk factors for mortality were evaluated using multivariable logistic regression. We identified 163 patients with IAC, compared to 161 with candidemia. Types of IAC were intra-abdominal abscesses (55%), secondary peritonitis (33%), primary peritonitis (5%), infected pancreatic necrosis (5%), and cholecystitis/cholangitis (3%). Eighty-three percent and 66% of secondary peritonitis and abscesses, respectively, stemmed from gastrointestinal (GI) tract sources. *C*. *albicans* (56%) and *C*. *glabrata* (24%) were the most common species. Bacterial co-infections and candidemia occurred in 67% and 6% of patients, respectively. Seventy-two percent of patients underwent an early source control intervention (within 5 days) and 72% received early antifungal treatment. 100-day mortality was 28%, and highest with primary (88%) or secondary (40%) peritonitis. Younger age, abscesses and early source control were independent predictors of survival. Younger age, abscesses and early antifungal treatment were independently associated with survival for IAC stemming from GI tract sources. Infectious diseases (ID) consultations were obtained in only 48% of patients. Consulted patients were significantly more likely to receive antifungal treatment. IAC is a common disease associated with heterogeneous manifestations, which result in poor outcomes. All patients should undergo source control interventions and receive antifungal treatment promptly. It is important for the ID community to become more engaged in treating IAC.

## Introduction

Invasive *Candida* infections are the most common non-mucosal fungal diseases among hospitalized patients in the developed world. Invasive candidiasis comprises candidemia and deep-seated candidiasis [1, 2]. Intra-abdominal candidiasis (IAC) is the most common type of deep-seated candidiasis, but remains poorly understood compared to candidemia [3]. Epidemiologic reports, antifungal treatment trials and diagnostic studies of invasive candidiasis have focused largely on candidemia [4]. Research on IAC is limited by the disease’s clinical heterogeneity, a lack of standardized disease definitions, and difficulties in establishing diagnoses [3, 5, 6].

IAC encompasses a range of disease manifestations, which occur in patients with various underlying conditions and risk factors involving the gastrointestinal (GI) tract and digestive system [6, 7]. The clinical significance of *Candida* in cultures of samples from intra-abdominal sites is controversial, as mixed infections with bacteria are frequent and the settings in which antifungal treatment is beneficial are not established conclusively [6, 8]. Clearly, *Candida* contributes to poor outcomes in some patients with intra-abdominal infections [7, 9]. However, other patients do well following source control and antibacterial treatment, without receiving antifungal agents.

Clinical studies of IAC and the development of well-formulated guidelines for its management are recognized as pressing priorities [3]. The objectives of this study were to determine the incidence of IAC compared to candidemia, define clinical characteristics and microbiology of the disease based on types and anatomic sources of infection, describe treatment and outcomes with particular attention to identifying risk factors for mortality, and assess the involvement of infectious diseases (ID) physicians in patient care.

## Methods

### Study design and definitions

We performed a retrospective study of adults diagnosed with IAC at the University of Pittsburgh Medical Center-Presbyterian University Hospital (UPMC-PUH) from January 1, 2012 through December 31, 2013. UPMC-PUH is a flagship academic, acute care, adult, medical-surgical referral, organ transplant and level 1 trauma center. The hospital has 586 acute medical-surgical and 156 ICU beds. Pediatric, obstetrical and gynecological patients, and most oncology patients are cared for at sister UPMC hospitals. The study was approved by the University of Pittsburgh Institutional Review Board. Informed consent was not given by participants for their clinical records to be reviewed. Patient information was anonymized and de-identified prior to the analysis.

Subjects were identified by reviewing microbiologic cultures that yielded *Candida* from intra-abdominal sources. IAC was defined in patients with clinical evidence of intra-abdominal infection and isolation of *Candida* in a sample collected from an intra-abdominal site collected under sterile conditions, in accordance with recently proposed criteria [3, 7]. We excluded patients whose samples were collected from drains that were in place >24 hours. We included only the initial episode of infection for each patient. The number of cases of IAC and candidemia included patients with the respective disease alone and patients who had both diseases. Our classification of IAC based on types and anatomic sources of infection is summarized in Table 1.

**Table 1:** Classification of types of intra-abdominal candidiasis (IAC).

IAC classification*	Definition
Primary peritonitis	Peritoneal inflammation** associated with recovery of *Candida* spp., occurring in the absence of an apparent breach of the GI tract or a pathologic process in a visceral organ.
Secondary peritonitis stemming from a GI tract source	Peritoneal *Candida* infection resulting from a pathologic process or breach of the GI tract (stomach, small bowel or colon), such as perforation, surgical leak or trauma.
Intra-abdominal abscess stemming from a GI tract source	Localized collection of *Candida* and pus that is walled-off from healthy tissue, resulting from a pathologic process or breach of the GI tract. Collections may be identified by imaging studies*** or intra-operatively.
Secondary peritonitis stemming from a hepatobiliary or pancreatic source	Peritoneal *Candida* infection resulting from a pathologic process of the liver, gallbladder, biliary or hepatic ducts, or pancreas.
Intra-abdominal abscess stemming from a hepatobiliary or pancreatic source	Abscess (as defined above) resulting from a pathologic process of the liver, gallbladder, biliary or hepatic ducts, or pancreas. Infected bilomas, pancreatic pseudocysts or other (peri)pancreatic collections are categorized as abscesses.
Infected pancreatic necrosis	*Candida* infection of non-vitalized pancreatic tissue resulting from chronic pancreatitis.
Cholecystitis, cholangitis	*Candida* infection of the gallbladder or biliary tract.

* In the classification scheme, sources of peritonitis and abscesses are divided into: a) gastrointestinal (GI) tract (stomach and intestines), and b) hepatobiliary system (liver, gallbladder, and associated ducts) or pancreas.

** Peritoneal inflammation was defined by neutrophil counts >250/mm^3^.

*** A majority of patients in this study received computed tomography scans.

The primary end-point was 100-day mortality, which was selected because complications of IAC may occur several weeks after diagnosis. Recurrent IAC was defined as culture-proven *Candida* infection occurring after an apparent resolution of clinical or radiographic findings of an initial *Candida* infection. Persistent IAC was defined as infection continuing for ≥48 hours after appropriate source control and active antifungal treatment, for which *Candida* was re-isolated on culture from an intra-abdominal sample. Appropriate source control was defined as adequate drainage of infected material and surgical correction of the underlying pathology (e.g. perforation or leak). Active treatment was defined by use of an appropriately dosed antifungal against an infection that: 1) was caused by an isolate that was susceptible to the agent *in vitro* (if testing was performed); 2) was caused by a *Candida* species that is not intrinsically resistant to the agent; and 3) occurred in a patient who was not previously exposed to the agent. Community- and hospital-acquired IAC and septic shock were defined by standard criteria [10, 11]. We defined an intervention as early if performed within 5 days of collecting the first culture-positive sample for *Candida*. We used 5 days as a cut-off to allow adequate time for the organism to grow in culture, and for patient care to be coordinated by the healthcare team.

### Statistical analysis

Descriptive statistics were used to summarize the cohort. Associations between categorical variables were analyzed using Pearson’s chi-square test or two-tailed Fisher’s exact test, as appropriate. Logistic regression was used to evaluate risk factors for mortality. Odds ratios with 95% confidence intervals were determined for each risk factor. Factors associated with mortality at the 0.1 significance level in univariate analysis were entered in the full multivariable model using a backward stepwise approach. Survival functions were estimated using the Kaplan-Meier method. STATA/SE software, version 13.1 (StataCorp, College Station, Texas) was used for statistical analysis.

## Results

### Clinical characteristics of IAC

In 2012–2013, 34,402 patients were admitted to UPMC-PUH. We identified 163 and 161 patients with IAC and candidemia, respectively (rates: 4.7 per 1,000 admissions). Overall, 25% (40/163) of patients with IAC were immunocompromised, and 53% (87/163) had abdominal surgery in the preceding 12 months (Table 2); 18% (30/163) presented in septic shock. The most common types of IAC were intra-abdominal abscesses (55%, 89/163) and secondary peritonitis (33%, 53/163), followed by primary peritonitis (5%, 8/163), infected pancreatic necrosis (5%, 8/163), and cholecystitis/cholangitis (3%, 5/163). Eighty-three percent (44/53) and 66% (59/89) of secondary peritonitis and abscesses, respectively, stemmed from gastrointestinal (GI) tract sources (Table 2). Clinical characteristics of secondary peritonitis and abscesses are compared in Table 3.

**Table 2:** Clinical characteristics, treatment and outcomes of patients with intra-abdominal candidiasis (IAC).

Parameter	All (n = 163)	Primary peritonitis (n = 8)	Secondary peritonitis, GI tract source (n = 44)	Abscess, GI tract source (n = 59)	Secondary peritonitis, hepatobiliary/pancreatic source (n = 9)	Abscess, hepatobiliary/pancreatic source (n = 30)	Infected pancreatic necrosis (n = 8)	Cholecystitis, cholangitis (n = 5)	*P* value
**Age**, median (range)	59 (21–90)	57 (40–78)	63 (23–90)	61 (23–84)	57 (32–65)	59 (21–88)	45 (26–74)	49 (41–85)	0.69
**Male gender**	89 (55)	6 (75)	22 (50)	25 (42)	7 (78)	18 (60)	7 (88)	4 (80)	0.06
**Race**^1^									
White	134/150 (89)	5/6 (83)	34/38 (89)	48/56 (86)	9/9 (100)	27/29 (93)	6/7 (86)	5/5 (100)	
Black	13/150 (9)	1/6 (17)	4/38 (11)	6/56 (11)	0/8	1/29 (3)	1/7 (14)	0/5	0.89
**Immunocompromised**^2^	40 (25)	2 (25)	11 (25)	12 (20)	7 (78)	8 (27)	0	0	0.01
Solid organ transplant	20 (12)	2 (25)	4 (9)	3 (5)	7 (78)	4 (13)	0	0	<0.001
**Obesity (BMI>30)**	51 (31)	3 (38)	11 (25)	18 (31)	2 (22)	13 (43)	2 (25)	2 (40)	0.72
**Surgery in the preceding 12 months**	87 (53)	1 (13)	19 (43)	42 (71)	7 (78)	14 (47)	1 (13)	3 (60)	<0.001
**Healthcare-associated IAC**	133 (82)	8 (100)	32 (73)	49 (83)	9 (100)	23 (77)	7 (88)	5 (100)	0.33
**Septic shock**	30 (18)	4 (50)	9 (20)	8 (14)	3 (33)	2 (7)	3 (38)	1 (20)	0.03
**Bacterial co-infection**	110 (67)	1 (13)	27 (61)	46 (78)	6 (67)	22 (73)	6 (75)	2 (40)	0.007
***C*. *glabrata* infection**	43 (26)	1 (13)	17 (39)	13 (22)	2 (22)	7 (23)	1 (13)	2 (40)	0.44
**Candidemia**^3^	8/125 (6)	0/6	1/32 (3)	2/44 (5)	2/9 (22)	2/24 (8)	1/7 (14)	0/3	0.34
**Type of source control**									
Surgical	96 (59)	2 (25)	40 (91)	29 (49)	6 (67)	11 (37)	7 (88)	1 (20)	<0.001
Percutaneous	59 (36)	0^4^	4 (9)	30 (51)	3 (33)	18 (60)	1 (13)	3 (60)	<0.001
Transgastric	2 (1)	0	0	0	0	1 (3)	0	1 (20)	0.04
**Antibacterial treatment**	161 (99)	8 (100)	43 (98)	58 (98)	9 (100)	30 (100)	8 (100)	5 (100)	1.00
**Antifungal treatment within 5 days**	117 (72)	7 (88)	33 (75)	37 (64)	9 (100)	23 (77)	7 (88)	2 (40)	0.15
**Recurrence/persistence**	11 (7)	0	2 (5)	6 (10)	2 (22)	1 (3)	0	0	0.44
**100-day mortality**	46 (28)	7 (88)	15 (34)	11 (19)	6 (67)	4 (13)	2 (25)	1 (20)	<0.001

Data are presented as absolute numbers (percentages), unless otherwise indicated.

^1^ Race was not available for all patients

^2^ Immunocompromised conditions: solid organ transplant (12%, 20/163), corticosteroid treatment (7%, 11/163), cancer chemotherapy (4%, 7/163), TNF-α blocker treatment (1%, 2/163)

^3^ Organisms recovered from blood: *C*. *albicans* (4), *C*. *glabrata* (2), *C*. *parapsilosis* (1), *C*. *krusei* (1)

^4^ Patients with primary peritonitis in the setting of liver cirrhosis underwent diagnostic, rather than therapeutic paracentesis

Abbreviations: GI, gastrointestinal; IAC, intra-abdominal candidiasis

**Table 3:** Comparison of clinical characteristics between patients with secondary peritonitis and intra-abdominal abscess.

Parameter	Secondary Peritonitis (n = 53)	Abscess (n = 89)	*P* value
**Age** in years, median (range)	60 (23–90)	59 (21–88)	0.95
**Male sex**	29 (55)	43 (48)	0.46
**Immunocompromised**	18 (34)	20 (23)	0.14
**Site of origin**^1^			
Gastric/Duodenum	10 (19)	16 (18)	0.89
Jejunum/Ileum	19 (36)	12 (14)	0.002
Colon	15 (28)	31 (35)	0.42
Liver/Gallbladder	6 (11)	12 (14)	0.71
Pancreas	3 (6)	17 (19)	0.03
**Prior intra-abdominal surgery**	26 (49)	56 (63)	0.11
**Healthcare-associated IAC**	41 (77)	72 (81)	0.61
**Septic shock**	12 (23)	10 (11)	0.07
**Perforation**^2^	29 (55)	42 (47)	0.39
**Bacterial co-infection**	33 (62)	68 (76)	0.07
***C*. *glabrata* infection**	19 (36)	20 (23)	0.08
**Candidemia**	3/4 (7)	4 (6)	1.00
**Recurrence/Persistence**	4 (8)	7 (8)	1.00
**100-day mortality**	21 (40)	15 (17)	0.003

Data are presented in absolute numbers (percentages), unless otherwise indicated.

^1^Site of origin in one female patient with intra-abdominal abscess was the genital tract.

^2^Rates of *Candida* infection by site of perforation during the study period were: stomach (39%, 12/31), duodenum (22%, 8/37), jejunum/ileum (25%, 17/69), colon (19%, 32/172), and appendix (4%, 2/50).

### Microbiology

A total of 180 *Candida* isolates were recovered (*C*. *albicans* 57%, C. *glabrata* 25%, *C*. *parapsilosis* 10%, *C*. *tropicalis* 5%, *C*. *krusei* 3%, *C*. *rugosa* 0.6% and *C*. *utilis* 0.6%). Infection with two *Candida* species occurred in 10% (17/163) of patients. Antifungal susceptibility testing was requested on 13% (23/180) of isolates (*C*. *albicans* 5, *C*. *glabrata* 13, *C*. *parapsilosis* 3, *C*. *krusei* 1, *C*. *rugosa* 1). All *C*. *glabrata* were susceptible dose-dependent to fluconazole (MIC range, 8–32 μg/ml); 38% (5/13) of isolates were recovered from patients with azole exposure in the preceding 3 months. Seventy-seven percent (10/13) and 23% (3/13) of *C*. *glabrata* were caspofungin-susceptible and -intermediate (MIC, 0.25 μg/ml), respectively. The latter isolates were associated with prior drug exposure. Non-*C*. *glabrata* isolates were azole- and echinocandin-susceptible.

Bacterial co-infection was present in 67% (110/163) of patients (S1 Table. Bacteria isolated in cases of co-infection). Enterococci were most common (29%, 47/163), followed by *Escherichia coli* (19%, 31/163) and *Klebsiella* spp. (15%, 24/163). *Candida* mono-infection was more likely during primary peritonitis (88%, 7/8) and cholecystitis/cholangitis (80%, 4/5) than other types of IAC (28%, 42/150; *p* = 0.001).

*C*. *glabrata* was significantly more likely to cause IAC among patients who had two or more prior abdominal surgeries within the previous year (47%, 14/30 *vs*. 22%, 29/133; *p* = 0.007), and to be involved in co-infections with multi-drug resistant (MDR) Gram-negative bacteria (extended-spectrum beta lactamase-producing or carbapenem-resistant *Enterobacteriaecae*) (14%, 6/43 *vs*. 2%, 2/120; *p* = 0.005). There were no differences in *Candida* spp. or rates of bacterial co-infection for healthcare-associated *vs*. community-acquired IAC, anatomic source, or presence of septic shock. There were trends toward more *C*. *glabrata* infections and bacterial co-infections during secondary peritonitis than in abscesses (*p* = 0.08 and 0.07, respectively).

At least one set of blood cultures was collected from 125 patients within 10 days of the diagnosis of IAC. The rate of candidemia was 6% (8/125). Twenty-four percent (30/125) of patients with IAC had concomitant bacteremia.

### Treatment and outcomes

Overall, 96% (157/163) of patients underwent at least one source control intervention (drainage and/or repair of an anatomical defect) to treat IAC. Early interventions (within 5 days of collecting the first culture-positive sample for *Candida*) were performed in 72% (118/163) of patients. Surgical intervention was undertaken in 59% (96/163), percutaneous drainage in 36% (59/163), and transgastric drainage in 1% (2/163). Reoperation was performed in 21% (20/96) of patients who underwent a surgical procedure, at median 9 days following the initial intervention (Interquartile range, IQR, 3–15 days). Secondary peritonitis was significantly more likely to be treated with abdominal surgery than were abscesses (87%, 45/52 *vs*. 45%, 40/89; *p*<0.001). Abscesses were more likely to undergo percutaneous drainage (53%, 48/90 *vs*. 13%, 7/52; *p*<0.001).

Ninety-nine percent (161/163) and 77% (125/163) of patients received antibacterial and antifungal therapy, respectively; 72% (117/163) received antifungal therapy within 5 days. Median duration of antifungal treatment was 14 days (range, 1–88 days). Early antifungal treatment was more likely among patients with healthcare-associated (75%, 100/133) than community-associated IAC (57%, 17/30; *p* = 0.05). Fifty-eight percent (94/163) of patients were treated with a single antifungal agent, including fluconazole (50%, 82/163), caspofungin (7%, 11/163) or voriconazole (0.6%, 1/163). Nineteen percent (31/163) of patients were treated with multiple agents; 29 patients received different agents sequentially, and 2 organ transplant patients received combination therapy with voriconazole and caspofungin due to concern for concomitant invasive aspergillosis.

**Fig 1:**
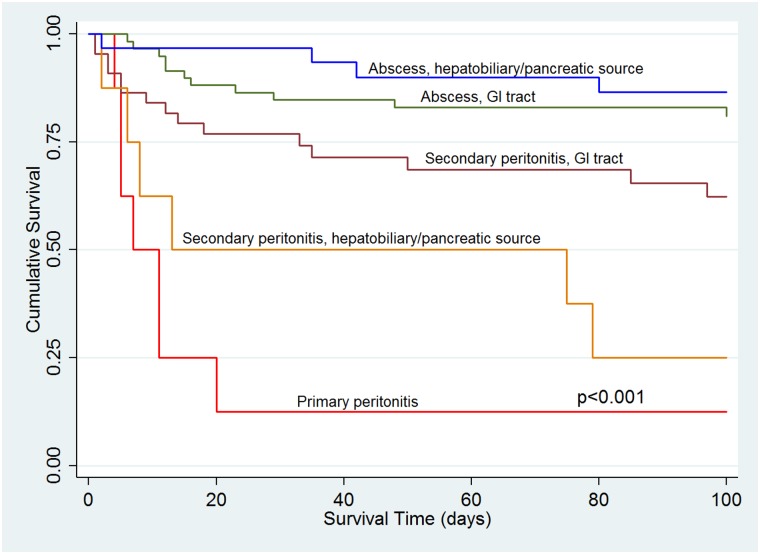
Survival analysis by type of intra-abdominal candidiasis.

Median length of hospital stay was 14 days after diagnosis of IAC (IQR, 7–26 days). Recurrent or persistent IAC occurred in 7% (11/163) of patients (S2 Table. Clinical characteristics of patients with culture-proven recurrent or persistent intra-abdominal candidiasis), 36% (4/11) of whom did not receive antifungal treatment initially. *C*. *glabrata* was the causative agent in 64% (7/11) of recurrent or persistent cases. The overall 100-day mortality rate was 28% (46/163), and highest among patients with primary peritonitis (88%, 7/8) and secondary peritonitis due to hepatobiliary/pancreatic (75%, 6/8) or GI tract sources (34%, 15/44) (Table 1, Fig 1). The 30-day mortality rate was 20% (32/163).

Younger age, the presence of an abscess and early source control were independently associated with survival among all patients with IAC (Table 4). There were no significant differences in mortality among patients with *Candida* mono-infection or bacterial co-infection, or among those with or without candidemia. Since IAC most commonly manifested as secondary peritonitis or abscesses that stemmed from GI tract sources, we also investigated factors associated with outcomes in this group (Table 5). Younger age, presence of an abscess and early antifungal therapy were independently associated with survival.

**Table 4:** Predictors of 100-day mortality for all subjects with IAC.

Parameter	Non-survivors* (n = 46)	Survivors (n = 117)	Univariate OR (95% CI)	Univariate *P* value	Multivariate OR (95% CI)	Multivariate *P* value
Age, median (interquartile range)	64 (57–77)	58 (46–67)	1.04 (1.01–1.06)	0.002	1.06 (1.03–1.09)	<0.001
Male sex	29 (63)	60 (51)	1.62 (0.80–3.26)	0.18		
Solid organ transplant	9 (20)	11 (9)	2.34 (0.90–6.10)	0.08	3.04 (0.98–9.43)	0.054
Obesity (BMI >30)	15 (33)	36 (31)	1.09 (0.52–2.26)	0.82		
Healthcare-associated disease	39 (85)	94 (80)	1.36 (0.54–3.44)	0.51		
APACHE II score, mean (range)	17 (7–29)	15 (3–29)	1.04 (0.96–1.12)	0.32		
Septic shock	15 (33)	15 (13)	3.29 (1.45–7.48)	0.004	…	…
Perforation	20 (43)	51 (44)	1.00 (0.50–1.98)	0.99		
Presence of abscess	15 (33)	74 (63)	0.28 (0.14–0.58)	0.001	0.25 (0.11–0.57)	0.001
Bacterial co-infection	26 (57)	84 (72)	0.51 (0.25–1.04)	0.06	…	…
*C*. *glabrata* infection	11 (24)	32 (27)	0.83 (0.38–1.84)	0.65		
Candidemia	3/36 (8)	5/89 (6)	1.53 (0.35–6.76)	0.57		
Surgical intervention	26 (57)	70 (60)	0.87 (0.44–1.74)	0.70		
Surgical intervention (within 5d)	19 (41)	58 (50)	0.51 (0.23–1.14)	0.34		
Source control intervention (within 5d)	25 (54)	93 (79)	0.38 (0.18–0.76)	0.002	0.23 (0.11–0.57)	0.001
Antifungal treatment (within 5d)	31 (67)	86 (74)	0.74 (0.36–1.56)	0.44		
Infectious disease consultation	21 (46)	58 (50)	0.85 (0.43–1.69)	0.65		

Data are presented in absolute numbers (percentages), unless otherwise indicated. Variables in ellipsis were removed from the multivariable analysis using backward elimination.

*Cause of death: sepsis (20), multi-organ failure (4), heart failure/cardiac event (4), respiratory failure (4), liver failure (2), hemorrhage/ischemia (2), hospice (7), unknown (3)

Abbreviation: OR, odds ratio.

**Table 5:** Predictors of 100-day mortality for subjects with IAC stemming from GI tract sources.

Parameter	Non-survivors (n = 26)	Survivors (n = 77)	Univariate OR (95% CI)	Univariate *P* value	Multivariate OR (95% CI)	Multivariate *P* value
Age, median (interquartile range)	68 (58–77)	58 (46–68)	1.04 (1.01–1.08)	0.01	1.04 (1.01–1.08)	0.018
Male	15 (58)	32 (42)	1.92 (0.78–4.72)	0.16		
Solid organ transplant	3 (12)	4 (5)	2.38 (0.50–11.43)	0.28		
Obesity (BMI >30)	11 (42)	18 (23)	21.84 (0.67–5.02)	0.07	…	…
Healthcare-associated disease	20 (77)	61 (79)	0.87 (0.30–2.54)	0.81		
APACHEII score	17 (7–27)	16 (5–29)	1.04 (0.94–1.14)	0.47		
Septic shock	6 (23)	11 (14)	1.8 (0.59–5.48)	0.30		
Perforation	19 (73)	51 (66)	1.38 (0.52–3.71)	0.52		
Presence of abscess	11 (42)	48 (62)	0.44 (0.18–1.09)	0.08	0.36 (0.13–0.96)	0.042
Bacterial co-infection	16 (62)	57 (74)	0.56 (0.22–1.44)	0.23		
*C*. *glabrata* infection	7 (27)	23 (30)	0.86 (0.32–2.34)	0.78		
Candidemia	0/19	3/57 (5)		0.57		
Surgical intervention	19 (73)	50 (65)	1.47 (0.55–3.92)	0.45		
Surgical intervention (within 5d)	15 (58)	42 (55)	1.55 (0.63–3.81)	0.78		
Source control intervention (within 5d)	17 (65)	63 (82)	0.49 (0.20–1.22)	0.09	…	…
Antifungal treatment (within 5d)	13 (50)	56 (73)	0.38 (0.15–0.94)	0.04	0.36 (0.13–0.96)	0.042
Infectious disease consultation	6 (23)	28 (36)	0.53 (0.19–1.46)	0.22		

Data are presented in absolute numbers (percentages), unless otherwise indicated. Variables in ellipsis were removed from the multivariate analysis using backward elimination.

Abbreviation: OR, odds ratio.

ID consultation was obtained in 48% (79/163) of patients. Median time to initial consultation was 2 days from the collection of the first sample that was culture-positive for *Candida* (IQR, -2 to +6 days). Consulted patients were more likely to receive antifungal treatment (82%, 65/79 *vs*. 60%, 50/84; *p* = 0.002). Forty-eight percent (35/79) of ID consults resulted in initiation of antifungal therapy.

## Discussion

Our study is notable for several important findings, beginning with the observation that IAC was at least as common as candidemia at our center. We demonstrate that IAC encompasses complex and highly heterogeneous types of infection, which were associated with 100-day mortality rates that ranged from 13% to 88%. Aggressive management was crucial for improved outcomes, as source control interventions and antifungal treatment within 5 days were independently associated with survival among the entire cohort and large subgroups of patients, respectively. Early antifungal treatment occurred in 72% of patients. In contrast, 99% of patients received antibiotic treatment, often in the absence of positive bacterial cultures. Taken together, our findings indicate that IAC is a disease associated with poor outcomes, and for which ID physicians often are not involved in patient care.

The heterogeneity of IAC poses a formidable hurdle to research. We developed a classification scheme based on the types and anatomic sources of infection, which is more precise than previous efforts to categorize IAC and correlates with patient outcomes [3, 12]. In this scheme, IAC manifests as primary peritonitis, secondary peritonitis, intra-abdominal abscesses, biliary infections, or infected pancreatic necrosis. Secondary peritonitis and abscesses, which account for a significant majority of IAC, are subdivided into infections that stem from either GI tract or hepatobiliary/pancreatic sources. Previous descriptions of IAC often used the term “secondary peritonitis” loosely to refer to both peritonitis and abscesses [3, 5, 13].

Abscesses, regardless of source, were associated with a relative low mortality rate (17%), whereas primary peritonitis and secondary peritonitis due to hepatobiliary/pancreatic or GI tract sources carried the highest mortality (88%, 75% and 34%, respectively). Patients with primary or secondary peritonitis were significantly more likely than patients with abscesses to have septic shock, a predictor of mortality in several studies [7, 9, 14, 15]. The combined mortality rate of 47% (28/60) for *Candida* peritonitis fell within the 25%-75% range reported previously [9, 15–21]. Our overall 100-day and 30-day mortality rates of 28% and 20%, respectively, were similar to the 27% rate at 30-days in a retrospective, multi-center study of IAC [7].

The finding that early source control was a crucial determinant of survival is consistent with previous data [7]. At the same time, our experience highlights the challenges in achieving timely and definitive source control. Twenty-five percent (39/157) of interventions occurred > 5 days after diagnosis, with delays often due to the severity of disease, complications of prior surgeries and/or lack of distinct foci of infection. Twenty-one percent of patients who were treated surgically required an additional intervention; 7% of patients were documented by rigorous criteria to have recurrent or persistent *Candida* infections. The superior survival we observed with abscesses likely reflects a greater likelihood of complete extirpation than with less well-localized infections. Not surprisingly, abscesses were significantly more likely than secondary peritonitis to be treated with percutaneous drainage, rather than surgical interventions.

The role of antifungal agents in the treatment of IAC is controversial [6–9, 18, 20, 22–25], and practice guidelines are conflicting [12, 26, 27]. Moreover, there is ongoing debate about the clinical significance of *Candida* recovered from intra-abdominal sites [8]. Therefore, it is notable that our data and those from a recent study establish the value of timely antifungal treatment in important subgroups of patients with IAC [7]. Mortality for *Candida* mono-infection and *Candida*-bacterial co-infections was comparable in each study, indicating that *Candida* spp. are significant pathogens within the abdominal cavity rather than innocent bystanders [8]. While it is clear that some patients with IAC do well without antifungal treatment, our findings suggest that clinicians are unable to accurately identify these patients. In many regards, the management of IAC at present resembles that of candidemia in the pre-fluconazole era, when *ad hoc* decisions were made about initiating antifungal treatment [28]. After it became apparent that clinicians were unable to accurately select those candidemic patients who required treatment, practice guidelines were changed to endorse an antifungal agent for all positive blood cultures [27, 28]. Likewise, we advocate immediate antifungal treatment for all patients with intra-abdominal infections in which *Candida* spp. are recovered from surgical samples or freshly-placed drains. Current data are insufficient to recommend a preferred agent for treatment of IAC. Several studies of candidemia suggest that time to initiation of treatment may be a more important determinant of outcome than choice of a specific antifungal [29–31]. In this regard, blood-based non-culture tests like β-D-glucan and polymerase chain reaction assays, used judiciously, may advance the management of IAC by diagnosing patients earlier than cultures of intra-abdominal specimens [1, 2, 32].

Carefully-coordinated, multi-disciplinary patient care is essential to improving outcomes for IAC [3, 6]. A recent editorial calls for interactions between intensivists, surgeons and radiologists, recognizes a need for standardized antimicrobial stewardship and source control protocols, but does not mention a role for ID practitioners [6]. In this light, the low rate of ID consultation in our study is perhaps not surprising. Most studies of IAC appear in critical care or surgical literature, while ID practice and studies on systemic candidiasis have focused largely on candidemia. In fact, blood cultures were positive for *Candida* in only 6% of our cases, a finding consistent with 0–32% rates in previous studies [15, 18–20, 32, 33]. We demonstrated an impact of ID involvement, as antifungal treatment was significantly more likely among patients who received a consultation. ID physicians were responsible for initiating an antifungal agent in 48% (35/79) of consults. Consultation was not associated with improved survival, but we cannot exclude biases in obtaining consults for more difficult cases.

Our finding that 67% of IAC were mixed infections is consistent with reports that bacteria, in particular enteric pathogens like *Enterococcus* and *Enterobacteriaceae*, are recovered with *Candida* in most cases [7, 15]. As in other studies, *C*. *albicans* was the most common species [7, 9, 15, 16, 34]. *C*. *glabrata*, the second most common species, was notable for significant associations with multiple prior abdominal surgeries and MDR Gram-negative bacterial co-infection. *C*. *glabrata* candidemia also has been linked to GI tract and biliary sources [35]. Interestingly, 10% of *Candida* isolates were *C*. *parapsilosis*, a species long associated with exogenous sources such as intravenous catheters. Emerging microbiome data now indicate that *C*. *parapsilosis* constitutes part of the GI flora in some patients [36].

We recently reported that IAC, in particular cases due to *C*. *glabrata*, was a hidden reservoir for emergence of echinocandin resistance [37]. Along these lines, it is notable that antifungal susceptibility testing was requested on only 30% of *C*. *glabrata* and 13% of all *Candida* isolates. One hundred percent and 23% of *C*. *glabrata* tested had reduced susceptibility to fluconazole and caspofungin, respectively. Other centers have reported lower rates of non-susceptibility during IAC [7], which may reflect differences in patient populations and/or antifungal usage. Our past and current data suggest that susceptibility testing should be performed routinely among patients with IAC who have remote or ongoing antifungal exposure [37].

It is important to acknowledge that our results were shaped by practices and populations at UPMC-PUH. For example, we care for many patients undergoing high-risk GI surgery and organ transplantation, but few pediatric, obstetrical-gynecological or oncology patients. Experience at other centers may differ, which highlights the importance of understanding patient populations, epidemiology, clinical manifestations and outcomes locally. It is also possible that we missed cases in which intra-abdominal infections were not drained or samples were not cultured. Likewise, the rates of IAC and recurrent or persistent infections were certainly under-estimated due to the rigorous definitions used in this study. Our ability to draw conclusions about certain IAC types (such as primary peritonitis, infected pancreatic necrosis and cholecystitis/cholangitis) was limited by the small number of cases. Thus, care in interpreting comparisons between IAC subgroups is warranted. Lastly, our study design precluded us from identifying risk factors for IAC, describing rates of disease among patients with various predisposing conditions, accurately determining the start date of infections, or assessing the efficacy of pre-emptive antifungal treatment among high-risk patients.

Further studies of IAC are needed to corroborate and expand upon our findings. Recent papers demonstrate that multicenter studies of deep-seated candidiasis are feasible, even among complex surgical patients [6, 7, 38]. Given the heterogeneity of IAC, studies should focus on well-defined subgroups of patients that share disease manifestations or predisposing conditions [6]. In this regard, our classification scheme (or variations) may be useful. At the same time, there is need for more research on the pathogenesis of IAC. Recently-developed mouse models of IAC demonstrated that *C*. *albicans* and *C*. *glabrata* gene expression and pathogenic mechanisms differed from those of hematogenous candidiasis [39, 40]. Animal models and other experimental systems are also powerful tools for studying diagnostics, antifungal treatment and emergence of resistance, in the absence of clinical trial data [3]. Finally, ID specialists have important roles to play in the management of IAC by guiding antifungal treatment, interpreting antifungal susceptibility patterns, integrating non-culture diagnostic tests into patient care, and designing stewardship protocols and research studies. It is incumbent upon the ID community to become more actively engaged in patient care and research into the disease.

## Supporting Information

S1 Table Bacteria isolated in cases of co-infection. (DOCX)

S2 Table Clinical characteristics of patients with culture-proven recurrent or persistent intra-abdominal candidiasis. (DOCX)
