# Supplementary material for: Intra-Abdominal Candidiasis: The Importance of Early Source Control and Antifungal Treatment
Source: PLoS One. 2016 Apr 28;11(4):e0153247. doi: 10.1371/journal.pone.0153247 (PMC4849645; doi:10.1371/journal.pone.0153247)
Supplement: S1 Table — (DOCX) [file pone.0153247.s001.docx]

**S1 Table. Bacteria isolated in cases of co-infection**

| **GRAM POSITIVE PATHOGENS (112)** | |
| --- | --- |
| Virdans group Streptococci  Group B Streptococcus  Group C Streptococcus  Microaerophilic Streptococci  *Streptococcus anginosus*  Non-hemolytic Streptococci, not group D  Pediococcus | 17  3  1  2  1  2  1 |
| *Enterococcus faecalis*  *Enterococcus faecium*  *Enterococcus avium*  *Enterococcus raffinosus* | 22  24 (VRE 22)  7  1 |
| *Staphylococcus aureus*  *Staphylococcus lugdunensis*  Staphylococcus species | 3  1  1 |
| Lactobacillus  Peptostreptococcus | 7  2 |
| Coagulase-negative Staphylococci  Diphtheroids  Eubacterium | 10  3  1 |
| *Clostridium innocuum*  *Clostridium perfringens*  *Clostridium subterminale* | 1  1  1 |

Abbreviations: CRE, carbapenem-resistant *Enterobacteriaecae*; ESBL, extended-spectrum beta lactamase-producing; VRE, vancomycin-resistant *Enterococcus*

| **GRAM NEGATIVE PATHOGENS (120)** | |
| --- | --- |
| *Escherichia coli*  *Klebsiella pneumoniae*  *Klebsiella oxytoca* | 31 (ESBL 6)  20 (CRE 2)  4 |
|  |  |
| *Proteus mirabilis*  *Proteus vulgaris*  *Morganella morganii*  *Serratia marcescens*  *Providencia stuartii*  *Enterobacter cloacae*  *Enterobacter corrodens*  *Enterobacter aerogenes*  *Enterobacter sakazzakii*  *Citrobacter freundii* | 1  1  1  1  1  9  2  1  1  1 |
| *Pseudomonas aeruginosa*  *Stenotrophomonas maltophilia* | 13  4 |
| *Haemophilus influenza*  *Haemophilus parainfluenzae*  Wolinella species | 1  1  1 |
| *Bacteroides fragilis*  *Bacteroides thetaiotaomicron*  *Bacteroides caccae*  *Bacteroides buccae*  *Bacteroides vulgatus*  *Bacteroides distasonis*  *Prevotella melaninogenica* | 8  4  3  2  3  1  4 |
| *Fusobacterium* species | 1 |
