# Supplementary material for: Intra-Abdominal Candidiasis: The Importance of Early Source Control and Antifungal Treatment
Source: PLoS One. 2016 Apr 28;11(4):e0153247. doi: 10.1371/journal.pone.0153247 (PMC4849645; doi:10.1371/journal.pone.0153247)
Supplement: S2 Table — (DOCX) [file pone.0153247.s002.docx]

**S2 Table. Clinical characteristics of patients with culture-proven recurrent or persistent intra-abdominal candidiasis**

| **Study Code** | **Age, Gender** | **Disease Description** | **Microbiology*** | **Initial Management** | **Complication/Outcome** |
| --- | --- | --- | --- | --- | --- |
| 3 | 69 F | Colon cancer s/p segmental colectomy, disruption of anastomosis c/w multiple intra-abdoninal abscesses | *C. glabrata*, VRE | Percutaneous drainage.  No antifungal treatment initially | Persistent abscesses. Fluconazole started on day 18. Died of septic shock on day 23. |
| 25 | 61 F | Acute diverticulitis c/w diverticular abscess | *C. tropicalis* | Percutanous drainage.  No antifungal treatment. | Recurrence of diverticular abscess. Surgical drainage on day 75. No antifungal. Survived. |
| 27 | 56 M | S/p kidney-pancreas transplant. Pancreatic necrosis, peripancreatic fluid collection | *C. albicans*,  *C. glabrata* | Percutaneous drainage.  Fluconazole prophylaxis changed to caspofungin on day 3. | Persistent fevers resolved after abdominal irrigation on day 9. Survived. |
| 41 | 44 M | Small bowel perforation in site of obstruction due to post lung transplant lymphoproliferative disorder. Secondary peritonitis. | *C. glabrata*,  *E. faecalis*, *Lactobacillus* | Small bowel resection with end jejunostomy.  Fluconazole treatment. | Developed intra-abdominal abscess. Died of ischemic enteritis with perforation on day 35. |
| 60 | 63 M | Gastrectomy for ischemia c/w gastric pouch staple line leak, intraabdominal abscess | *C. albicans*,  *C. glabrata* | Required Roux-en-Y esophagojejunostomy.  No antifungal treatment initially. | Anastomotic failure with recurrent leak and abdominal contamination necessitating surgical management Fluconazole started on day 90. Died of septic shock on day 100. |
| 65 | 59 F | Recurrent sigmoid diverticulitis c/w diverticular abscess | *C. albicans*,  *E. faecalis* | Percutaneous drainage.  Fluconazole started on day 5. | Recurrent diverticulitis. Infection cleared after surgical drainage on day 95. Survived. |
| 79 | 56 M | S/p kidney-liver transplant. Peripancreatic fluid collection | *C. albicans*,  VRE | Debridement of necrotic pancreas.  Caspofungin treatment. | Persistent infection despite surgical and percutaneous drainage. Died of sepsis on day 79. |

| 84 | 70 F | Sigmoid colon cancer with perforation c/w pelvic abscess | *C. glabrata*,  MRSA,  *E. faecalis*,  *B. vulgatus* | Percutaneous drainage.  No antifungal treatment initially. | Recurrent perforation on day 24. Surgical drainage of multiple intra-abdominal abscesses. Caspofungin treatment. Survived. |
| --- | --- | --- | --- | --- | --- |
| 136 | 59 M | Mesenteric ischemia due to thrombosed aortomesenteric bypass graft c/w graft infection, abscess cavity | *C. albicans*, VRE | Partial explantation of graft. Caspofungin treatment. | Persistent infection due to leak, required multiple abdominal irrigations. Survived. |
| 137 | 73 F | Pancreatic adenocarcinoma s/p Whipple c/w biliary leak, perihepatic abscess | *C. albicans*,  *C. glabrata*, ESBL *E. coli* | Percutaneous drainage.  Fluconazole treatment. | Recurrent abscess, *E. coli* bacteremia and *C. glabrata* fungemia on day 67. Died of septic shock on day 80. |
| 157 | 33 M | Intestinal transplant s/p multiple exploratory laparotomies for recurrent intestinal fistulas, infected blood clots | *C. glabrata*,  VRE | Multiple laparotomies for irrigation and drainage.  Caspofungin treatment. | Chronic graft rejection with recurrent leak and *C. glabrata* peritonitis. Graft enterectomy on day 63. Died of multiorgan failure on day 97. |

Antifungal susceptibilities were not performed on isolates that were recovered at the time of initial diagnosis.

Abbreviations: M, male; F, female; s/p, status post; c/w, consistent with; ESBL, extended-spectrum beta lactamase-producing; MRSA, methicillin-resistant *Staphylococcus aureus*; VRE, vancomycin-resistant *Enterococcus*; c/w, complicated with.
